# Earth-shaking J. LEAGUE supporters

**DOI:** 10.1186/s40623-022-01686-3

**Published:** 2022-08-09

**Authors:** Suguru Yabe, Kiwamu Nishida, Shinichi Sakai

**Affiliations:** 1 Geological Survey of Japan, National Institute of Advanced Industrial Science and Technology (AIST), Tsukuba Central 7, 1-1-1 Higashi, Tsukuba, Ibaraki 305-8567 Japan; 2 Earthquake Research Institute, The University of Tokyo, 1-1-1 Yayoi, Bunkyo-ku, Tokyo, 113-0032 Japan

**Keywords:** Human activity, J. LEAGUE, Seismic wave propagation, Urban seismology, Shallow subsurface structure

## Abstract

We conducted temporary seismic observations at the Hitachi-Kashiwa Soccer Stadium on a J. LEAGUE game day to obtain unique seismic records due to the collective action (i.e., jumping) of supporters, which were also recorded in a permanent Metropolitan Seismic Observation network (MeSO-net) station. This study investigated seismic wave excitation as well as seismic wave propagation from the stadium to its surroundings. The rhythms of the jumps of the supporters were characterized by analyzing audio data recorded in the stadium, which were compared with the characteristic frequencies observed in the seismic records. The characteristic frequencies in the seismic records are integer multiples of the jumping rhythms, which is consistent with the loading model of jumping people proposed in earlier studies. This implies that seismometers could be useful for monitoring collective human activity. Travel times were studied using deconvolved waveforms because seismic waves generated by the supporters are sinusoidal with vague onset. Polarization analysis was performed to measure the amplitude and polarization azimuths. The observed seismic wave propagation was compared with synthetic waveforms calculated using one-dimensional physical properties based on the Japan Seismic Hazard Information Station (J-SHIS). The synthetic waveforms calculated with the shallow and deep layer combined model are more consistent with observations of travel times and amplitude decay than those calculated with the only deep layer model, although a part of the observations cannot be explained by both models. This result suggests that the subsurface structure of J-SHIS is good in this region, although a more detailed three-dimensional structure and topography must be considered to fully explain the observations. As human-induced seismic signals are expected to be generated in various situations, this study shows that such unique seismic waves can be used as an artificial seismic source for validating and improving local shallow subsurface structural models in urban environments.

**Graphical Abstract:**

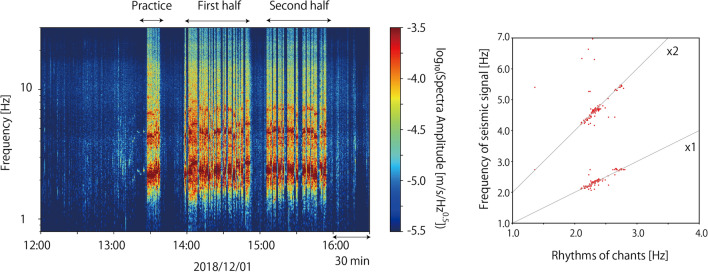

## Introduction

Globally, football fans cheer their teams, shout for goals, and jump for wins, and Japan is no exception. J. LEAGUE, a top football league in Japan, began in 1993 with ten teams. Currently, the number of teams has increased to 57 across Japan in 2021. Each team has enthusiastic fans who sing and jump in stadiums to support their teams and players, which literally causes shaking of the earth. With recent advances in urban seismology, there is an increase in the seismic records of urban environments. Such observations have captured seismic signals resulting from football games. For example, Euler et al. (2007) reported “footquakes” in Cameroon. They recorded strange seismic signals in their temporary seismic networks. The signals were composed of high-frequency (> 1 Hz) tremors, which was observed at the same time (i.e., no travel time) in seismic stations of the country. Finally, it was found that they were generated by people watching football games on television. Diaz et al. (2017) also reported footquakes at the FC Barcelona Stadium in Spain. Spiky signals were observed when FC Barcelona scored a goal.

In Japan, the Metropolitan Seismic Observation Network (MeSO-net) (National Research Institute for Earth Science and Disaster Resilience 2021) was deployed in the urban environment of the Tokyo metropolitan area (Sakai and Hirata 2009; Aoi et al. 2021). MeSO-net consists of more than 300 seismic stations located at the bottom of shallow boreholes at a depth of ~ 20 m to reduce cultural noise (Kasahara et al. 2009). As they are located near intensive human activities in Tokyo, they record seismic signals generated by various human activities, such as mobility and aircraft, as well as natural phenomena, such as earthquakes and thunders (Kawakita and Sakai 2009). In 2020, due to the COVID-19 pandemic, the noise levels of seismometers globally have decreased (e.g., Lecocq et al. 2020). A clear drop in the seismic noise level due to reduced social activities was also observed in the MeSO-net (Yabe et al. 2020; Nimiya et al. 2021).

**Fig. 1:**
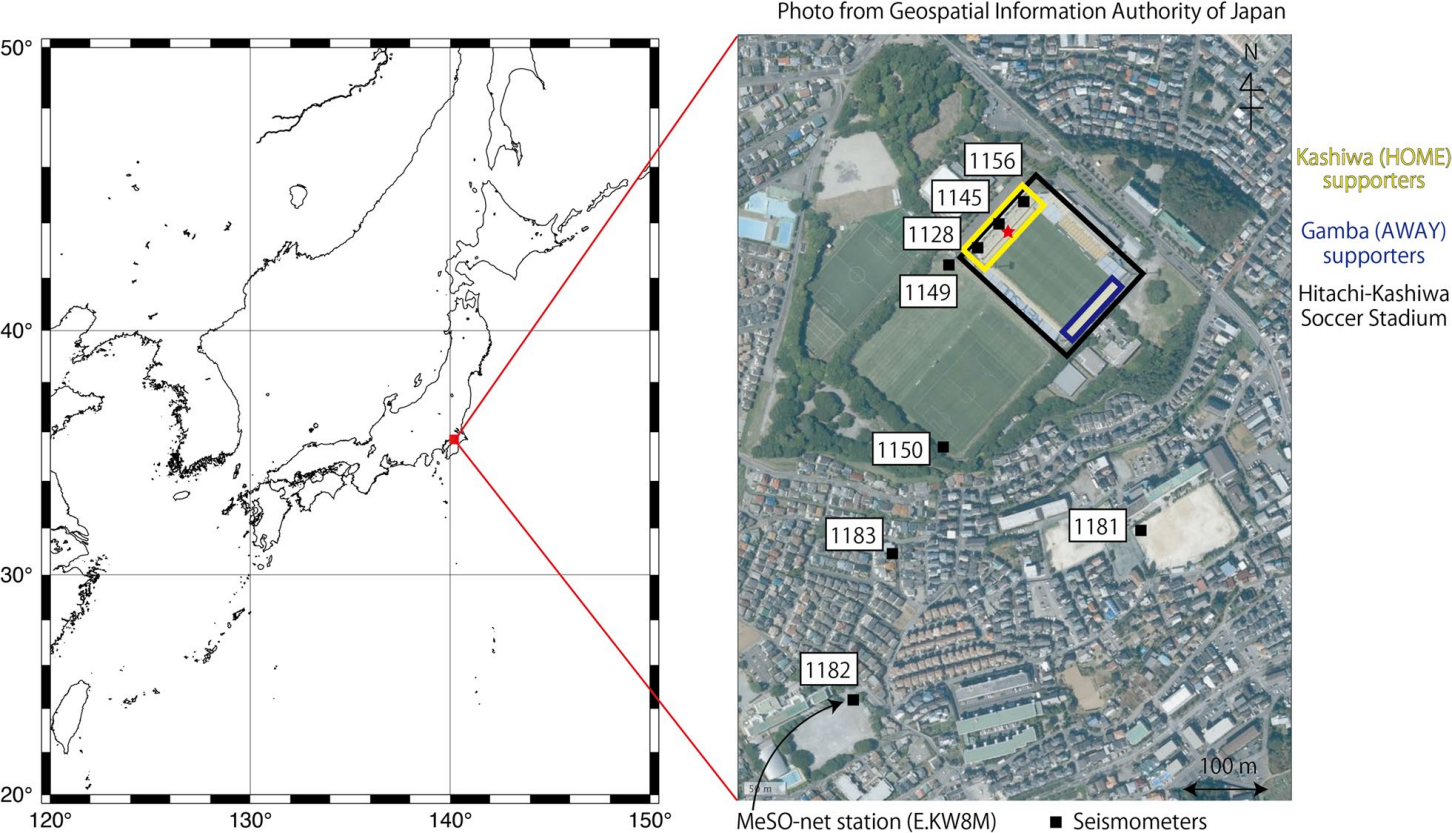
A station map around the Hitachi-Kashiwa Soccer Stadium. A small red star in the stadium is the assumed source location in synthetic wave calculations. A photograph was taken from Geospatial Information Authority of Japan (https://maps.gsi.go.jp/#5/36.104611/140.084556/; 2022/03/31 accessed)

**Fig. 2:**
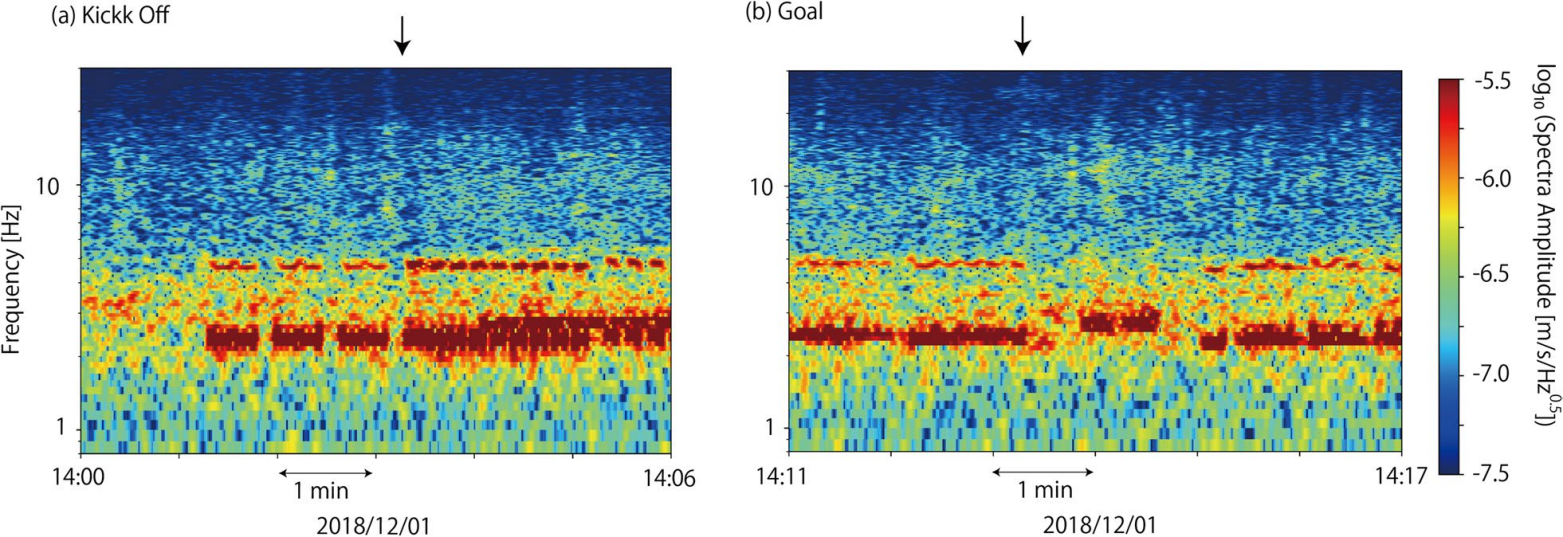
Example plots of running spectra for seismic signals (UD component) from J. LEAGUE games at Hitachi-Kashiwa Soccer Stadium observed at MeSO-net station E.KW8M. Arrows in figures represent timings of **a** kick-off and **b** goals, respectively

Among several J. LEAGUE teams located in Tokyo metropolitan area, ‘Kashiwa Reysol’ have their home stadium ‘Hitachi-Kashiwa Soccer Stadium (SANKYOFRONTIER Kashiwa Stadium)’ at approximately 600 m northeast of MeSO-net station E.KW8M (Fig. 1). By checking the seismic records of the E. KW8M station on a J. LEAGUE game day, we were able to find the characteristic signals (Fig. 2). The signals lasted long time; more than tens of seconds to minutes and repeated during the game. They had several characteristic frequencies, which changed over time (Fig. 2a). However, the signals become weak when a goal was scored (Fig. 2b). These signals appear different from those of the footquakes reported by Diaz et al. (2017), which are spiky when goals are scored. The long-lasting characteristic of J. LEAGUE signals is similar to the seismic signals generated by a rock concert at the stadium of FC Barcelona (Diaz et al. 2017). Seismic signals during the concert had characteristic frequencies according to songs and lasted during each song. We hypothesized that long-lasting signals observed in the J. LEAGUE would be generated by the supporters who constantly keep jumping and singing chants to cheer up their teams during the games.

This study conducted temporary seismic observations in and around the Hitachi-Kashiwa Soccer Stadium on a J. LEAGUE game day to understand seismic wave excitation in the stadium and its propagation from the stadium to its surroundings. We show that characteristic frequencies and amplitude of the observed seismic waves can be related to chants supporters were singing and the number of jumping supporters in the stadium, respectively. This suggests that observing human-induced seismic waves will be useful for monitoring human activity in a stadium. As shown by the noise level decrease during the COVID-19 pandemic (e.g., Lecocq et al. 2020; Yabe et al. 2020; Nimiya et al. 2021), seismometers can be a new tool for monitoring human activities in society. Observing and analyzing unique signals generated in urban environments will also be useful for assessing local shallow subsurface structural models (e.g., Quiros et al. 2016; Lavoué et al. 2020), which is key for improving ground shaking prediction. In the Tokyo metropolitan area, the Japan Seismic Hazard Information Station (J-SHIS) has constructed a detailed three-dimensional subsurface model (Fujiwara et al. 2009; 2012; National Research Institute for Earth Science and Disaster Resilience 2019). The subsurface structure was created with a ~ 1-km mesh, and the S-wave velocity variations for the top soil were created with a 250-m mesh. This model was constructed based on various structural explorations and geological knowledge, as well as a horizontal-to-vertical spectral ratio. Ground-motion simulations were also conducted to validate the model. We tested the J-SHIS structural model in the region by comparing our small-scale observation data with synthetic seismograms. We consider that this study presents a good pilot trial to use seismic waves induced by collective actions of people as an artificial seismic source for subsurface structure analysis. As this kind of human-induced seismic waves could be excited in various situations in urban environments, similar or more sophisticated analysis could be applicable everywhere in the world.

The rest of this article is organized as follows. We outline our temporary observations in the next section. In “Earth-shaking J. LEAGUE supporters” section, we show the relationship between actions of the supporters and ground shaking by investigating seismic data recorded just beneath the seats of the supporters and audio data recorded in the stadium. The results showed that the observed seismic waves were closely correlated with the rhythms of the chants of the supporters. In “Seismic wave propagation” section, we describe how the propagation of the generated wave using the seismic records of our temporary stations around the stadium and a MeSO-net station. In “Synthetic wave calculation” section, we calculate synthetic waveforms based on the shallow subsurface structure constructed by J-SHIS, and show that the calculated waveforms partly explain the observed features. In “Conclusions” section, we conclude that the unique seismic signals generated by human activities are useful for monitoring collective human activities and for validating and improving the regional shallow subsurface structure model in the urban environment.

## Temporary seismic observation on a J. LEAGUE game day

Our temporary seismic observations were conducted on December 1st, 2018, when the last game of the 2018 season was held at Hitachi-Kashiwa Soccer Stadium played between Kashiwa Reysol and Gamba Osaka. The game was kicked off at 14:03. Kashiwa Reysol scored in the 11th, 45th, 47th, and 63rd mins, while Gamba Osaka scored in the 28th and 79th mins, which resulted in a 4–2 win of Kashiwa Reysol. The total number of attendees was 13,067. The most energetic supporters of Kashiwa Reysol were seated in the western stand, whereas supporters of Gamba Osaka were seated in the eastern stand (Fig. 1).

We deployed eight seismometers in and around the Kashiwa-Hitachi Soccer Stadium on that day (Fig. 1). The seismometers were Lennartz 3D Lite Mark II. Three of them (Station 1156, 1145, and 1128 from north to south) were in the stadium nearby supports of the steeped stadium floor for Kashiwa Reysol supporters. Two of them (Station 1149 and 1150) were in the Hitachi-Kashiwa general ground, which is located south of the stadium. Station 1183 is located in the residential area between the stadium and MeSO-net station. Station 1182 is located on the ground surface just above the MeSO-net station. Station 1181 is located in a junior high school southeast of the stadium. Placements of seismometers were completed before 10:30, and their removal started after 17:00, which is approximately an hour after the game. In this study, we analyzed the data from 12:00 to 16:30. We also recorded audio in the stadium during the game to determine the action of the supporters.

The seismic records at temporary stations are velocity seismograms, whereas those at the MeSO-net station are acceleration seismograms. The acceleration seismogram was converted into velocity seismograms using numerical integration. We also removed the instrumental response for the seismograms of the temporary stations. The instrumental response of the MeSO-net stations was flat down to the DC component (Kasahara et al. 2009).

## Earth-shaking J. LEAGUE supporters

Seismic signals observed at the MeSO-net station during the game have several characteristic frequencies and continue from several tens of seconds to several minutes (Fig. 2). To understand the excitation of seismic waves caused by the supporters, we first checked the seismic records at Station 1145, which is located nearby supports of the steeped stadium floor for Kashiwa Reysol supporters.

**Fig. 3:**
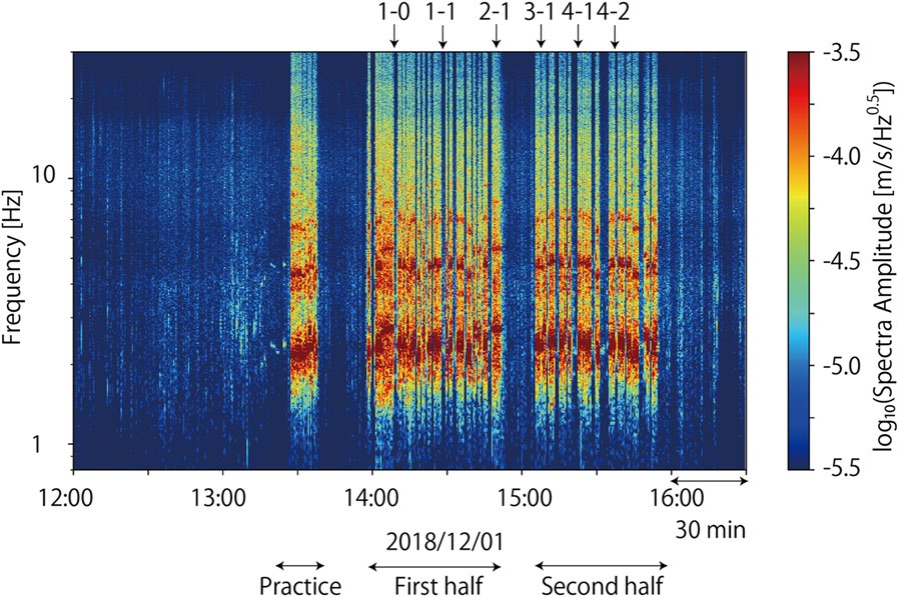
A running spectra plot of UD component of Station 1145

**Fig. 4:**
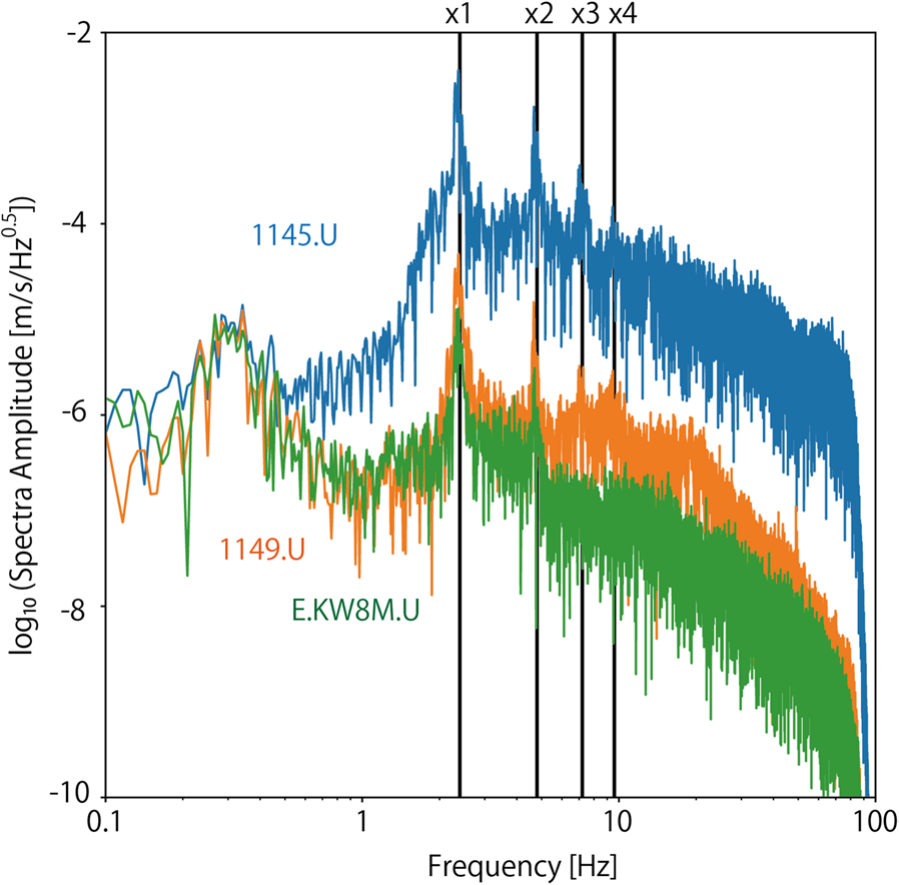
Spectra plots of UD components at Stations 1145, 1149, and E.KW8M between 14:30 and 14:32. A black vertical line “ × 1” represents the lowest characteristic frequency observed at the two stations, which is 2.4 Hz. Black vertical lines “ × 2”, “ × 3”, and “× 4” epresents two to four multiples of the lowest characteristic frequency (i.e., 4.8, 7.2, and 9.6 Hz, respectively)

Figure 3 shows the running spectra of the UD components at Station 1145. The amplitudes of the seismic signals were very high during the game and dropped at half time. They were also high before the kick-off. This time corresponds to when the players practiced in the field. Supporters started singing and jumping even before the game started. Figure 4 compares the spectra of the UD components at Stations 1145, 1149, and E.KW8M from 14:30 to 14:32. Two characteristic frequencies were observed for E.KW8M, whereas four characteristic frequencies were observed at station 1145 and 1149. The lowest characteristic frequency among these was 2.4 Hz, which was coherently observed at all stations. The other characteristic frequencies are integer multiples of the lowest characteristic frequency. Peaks were observed at 4.8, 7.2, and 9.6 Hz at 1145 and 1149, whereas only a peak at 4.8 Hz was observed at E.KW8M.

Ellis and Ji (2004) modeled loading caused by jumping people. According to them, a single force loading on the surface owing to a constant-rate jumping individual can be written as follows:

1 $$F\left(t\right)=W\left[1.0+ \sum_{n=1}^{\infty }{c}_{n} sin\left(\frac{2n\pi }{T}t+{\varphi }_{n}\right)\right],$$

where *W* is the weight of a person, *c*_*n*_ is the dynamic loading factor, *T* is the jumping period, and ${\varphi }_{n}$ is the phase lag. This suggests that integer multiple characteristic frequencies should be observed in seismic records when people are jumping. Actually, we observed integer multiple characteristic frequencies in seismic records during the game as shown in Fig. 4. Therefore, the observed seismic signals are expected to be induced by jumping supporters, not other noise signals.

We tested this hypothesis by comparing the seismic records with the audio recorded in the stadium during the game. We considered that the jumping periods of supporters were controlled by the rhythm of the chants they were singing, because some supporters hit drums at the center of supporters to lead singing chants, which works as a metronome for singing and jumping supporters. Hence, we measured the rhythm of the chants from the audio records and compared it with the characteristic frequencies in the seismic records.

**Fig. 5:**
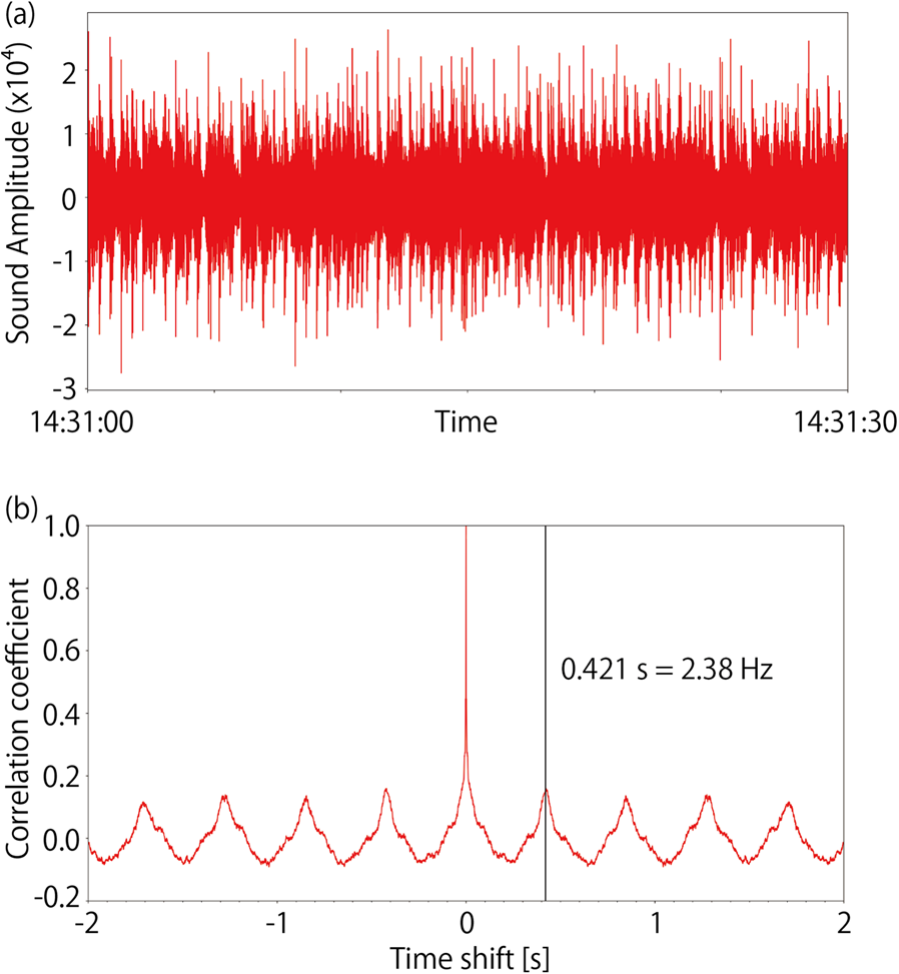
Plots of audio data for chants rhythm measurements. **a** 100–200 Hz band-passed waveform of sound record at 14:31:00 – 14:31:30. **b** Autocorrelation function of the envelope waveform converted from **a**. A vertical black line represents periodicity of the autocorrelation function

The rhythms of the chants were measured using the autocorrelation function of the envelope waveform of audio records. The audio records were band-passed between 100 and 200 Hz and converted to an envelope waveform, which was then low-pass filtered below 4 Hz. A bandpass filter was applied before constructing the envelope waveform to emphasize the sounds of the drums. A low-pass filter was applied to smooth the envelope waveforms. Examples of autocorrelation functions of the processed envelope functions are shown in Fig. 5. The audio record shown in Fig. 5 corresponds to the seismic record shown in Fig. 4. The calculated autocorrelation function has periodic peaks, which we define as the chant rhythm. In this case, the periodicity of the autocorrelation function was 0.421 s, which corresponds to a characteristic frequency of 2.38 Hz. This value agrees with the characteristic frequency of 2.4 Hz obtained from the seismic record shown in Fig. 4. We selected the largest peaks in the calculated autocorrelation function between lag times of 0.2 and 0.8 s (Fig. 5b), and rejected the picked frequencies if the widths of the peaks, where the correlation coefficients drop by 0.1 from the peak value, are larger than 0.2 s.

**Fig. 6:**
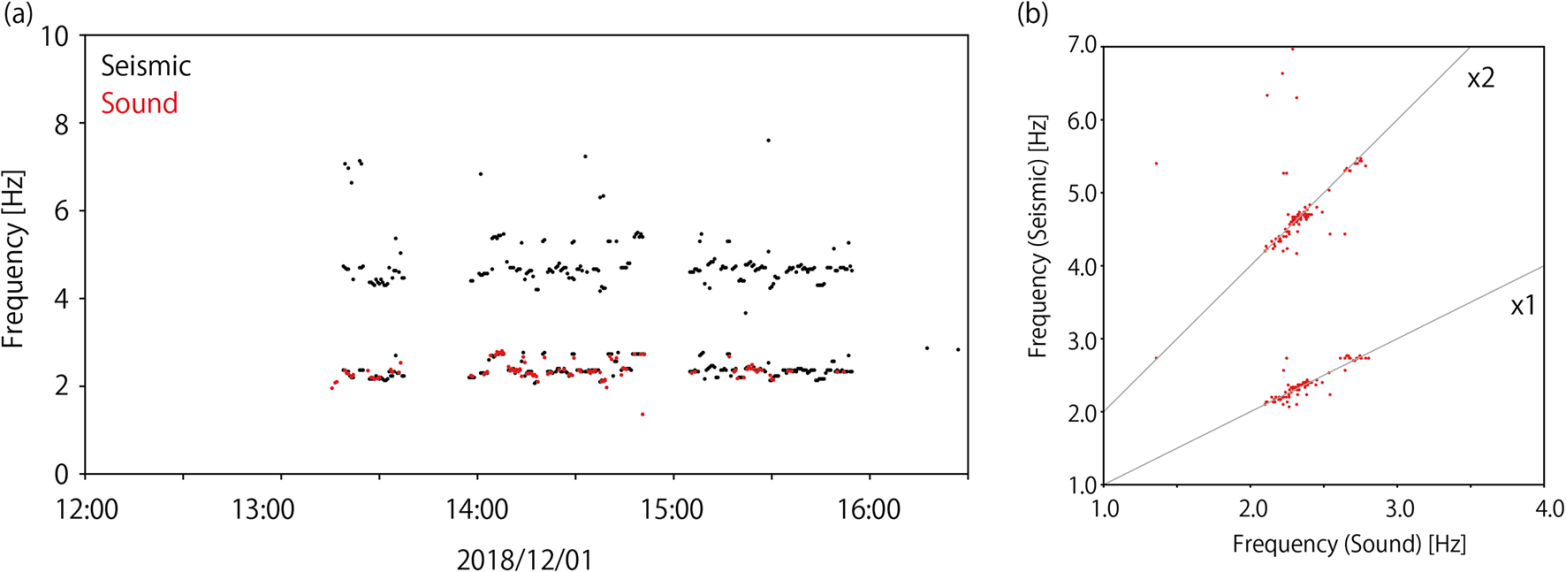
Scatter plots showing the comparisons of characteristic frequencies observed in seismic and sound records. **a** Time plot of observed characteristic frequencies. Black and red dots represent characteristic frequencies read from seismic and sound records, respectively. **b** Comparisons of two characteristic frequencies observed at the same timing. Gray lines represent one and two times multiple of chants rhythms

We made this comparison for the entire dataset used to investigate the relations between characteristic frequencies in seismic and audio records. The characteristic frequencies of the seismic records were measured using polarization analysis (Park et al. 1987; Yabe et al. 2020). The seismic waves generated by supporters are highly polarized, and polarization analysis is useful for extracting such features. Using time windows of 30 s shifted every 1 s, we calculated Fourier spectra of three-component seismograms. For every frequency, Fourier spectra covariance matrices were calculated, which were then stacked every 30 s. The stacked Fourier spectra covariance matrices are used to calculate the polarization amplitude and azimuths at every frequency and characteristic frequencies of the seismic signals. The polarization amplitude was defined as the maximum eigenvalue of the stacked covariance matrix. The polarization azimuths were calculated using a complex eigenvector for the maximum eigenvalue, which are along the direction in which the polarization dips. We also calculated singularity values defined as ${\left(3t\left({S}^{2}\right)-{t\left(S\right)}^{2}\right)}\!\left/ \!{2{t\left(S\right)}^{2}}\right.$ (Koper and Hawley 2010), where *S* is the stacked covariance matrix at a specific frequency and *t()* is the trace of a matrix. Larger singularity values mean that the polarization is more evident. The signal-to-noise ratio of every time window was calculated as well with the definition of the noise level as the log-averaged polarization amplitude between 12:00 and 13:00 h. The characteristic frequencies of seismic records were defined as frequencies at polarization amplitude peaks with signal-to-noise ratios larger than 10 and singularity values stacked for all stations larger than 0.8. The threshold of singularity values was selected so that the polarization is clearly observed in all stations. Figure 6 shows a comparison of the two characteristic frequencies read from the seismic and audio records. When the characteristic frequencies were observed, the timings were highly consistent with each other (Fig. 6a). It is also confirmed that the characteristic frequencies of the seismic records are integer multiples of the characteristic frequencies of the audio records (Fig. 6b). This observation is consistent with the model of Ellis and Ji (2004), which was explained above. Therefore, based on this result, we confirm that the seismic source of footquakes in Hitachi-Kashiwa Soccer Stadium is the earth-shaking J. LEAGUE supporters.

Good coincidences between characteristic frequencies in seismic and audio records provide us a good example of monitoring collective human activities through seismic waves. Figure 6a shows that characteristic frequencies varied with time, which represents supporters sang different chants during the game. It means that we can distinguish which chants supporters are singing only from seismic waves if we have a priori information on rhythms of supporters’ chants.

## Seismic wave propagation

In this section, we investigate the seismic wave propagation generated by the earth-shaking J. LEAGUE supporters. Using seismic records obtained in and around the stadium, we analyzed the travel time, spatial decay of amplitude, and polarization azimuth of the seismic waves.

**Fig. 7:**
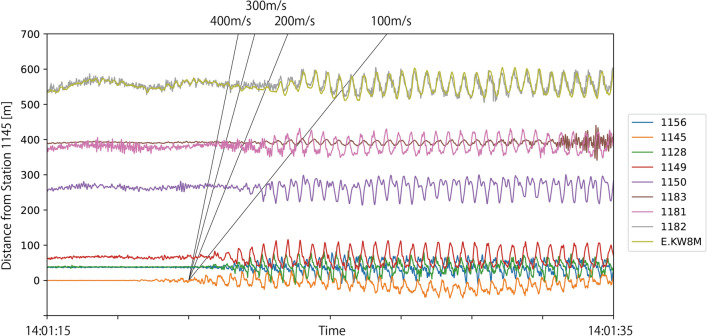
Plot exhibiting the close-up UD-component waveforms at all stations. Amplitudes are normalized at each station by the maximum value in the time window. Black lines represent travel times with constant velocity for references

**Fig. 8:**
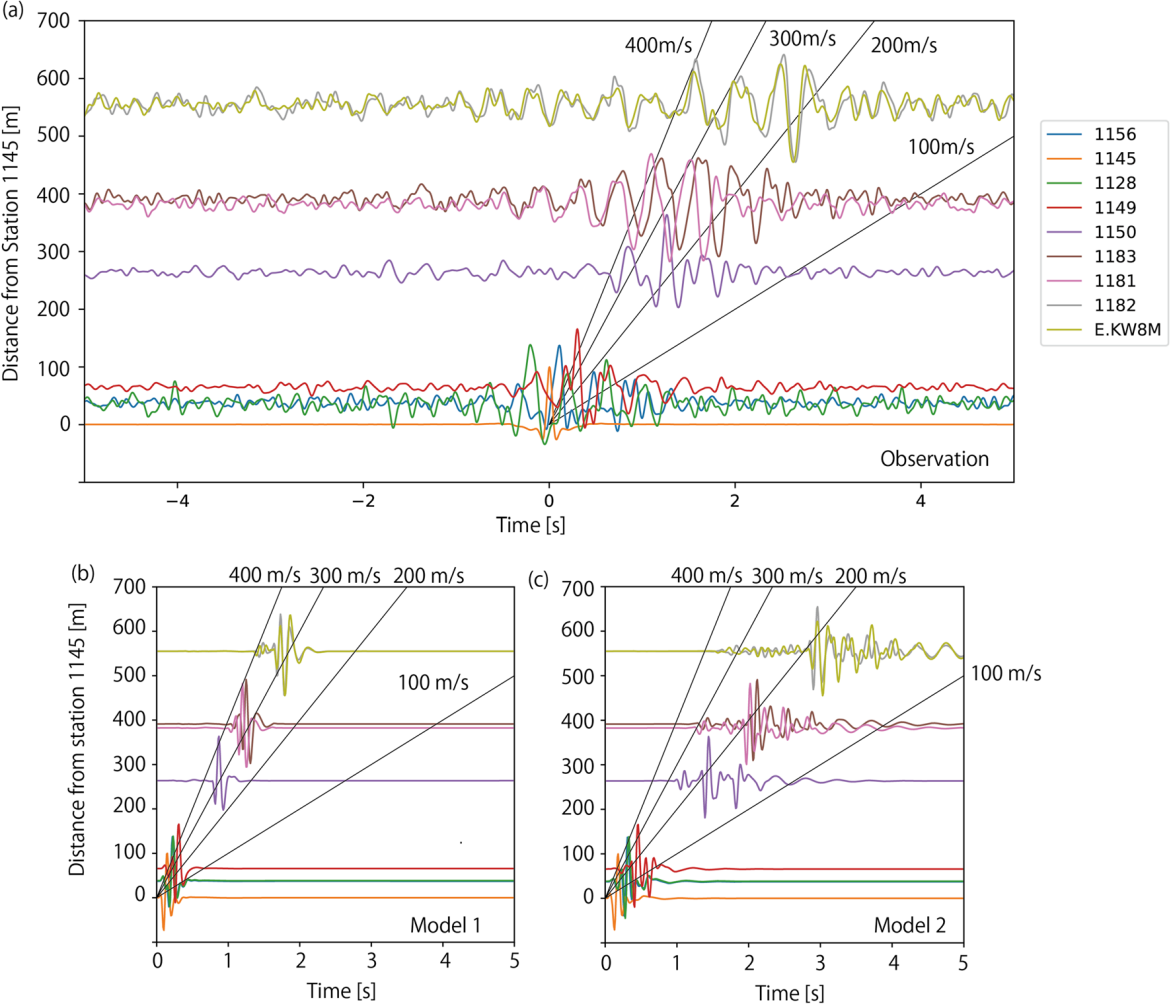
Travel time plot of observed and synthetic waveforms. **a** Deconvolved waveforms calculated from observed seismograms. UD components of velocity waveforms calculated for **b** Model 1 and **c** Model 2. Black lines represent travel times with constant velocity for references

First, we investigated the travel times of seismic waves. In the case of regular seismic signals, travel times are calculated by focusing on specific phases such as P- or S-waves. However, the seismic signals generated by supporters are sinusoidal, and their onset is vague (Fig. 7), which makes it difficult to calculate the travel time by picking phases. Instead, we calculated waveforms at each station deconvolved by waveforms at Station 1145 to emphasize the traveling isolated wave packets (Snieder and Şafak 2006; Nakata and Snieder 2011, 2012a, 2012b). We used time windows of 20 s and UD-component seismic waveforms when the characteristic frequencies were detected as discussed in the previous section. The calculated deconvolved waveforms were normalized by the maximum absolute value and stacked for all timings. The deconvolved waveforms obtained in Fig. 8a have clearer traveling wave packets than the original waveforms in Fig. 7. The largest amplitude propagates at approximately 200 m/s at stations 1150, 1182, and MeSO-net station, though onsets at stations 1181 and 1182 seems to be faster. Earlier and later dispersed waveforms are also observed.

**Fig. 9:**
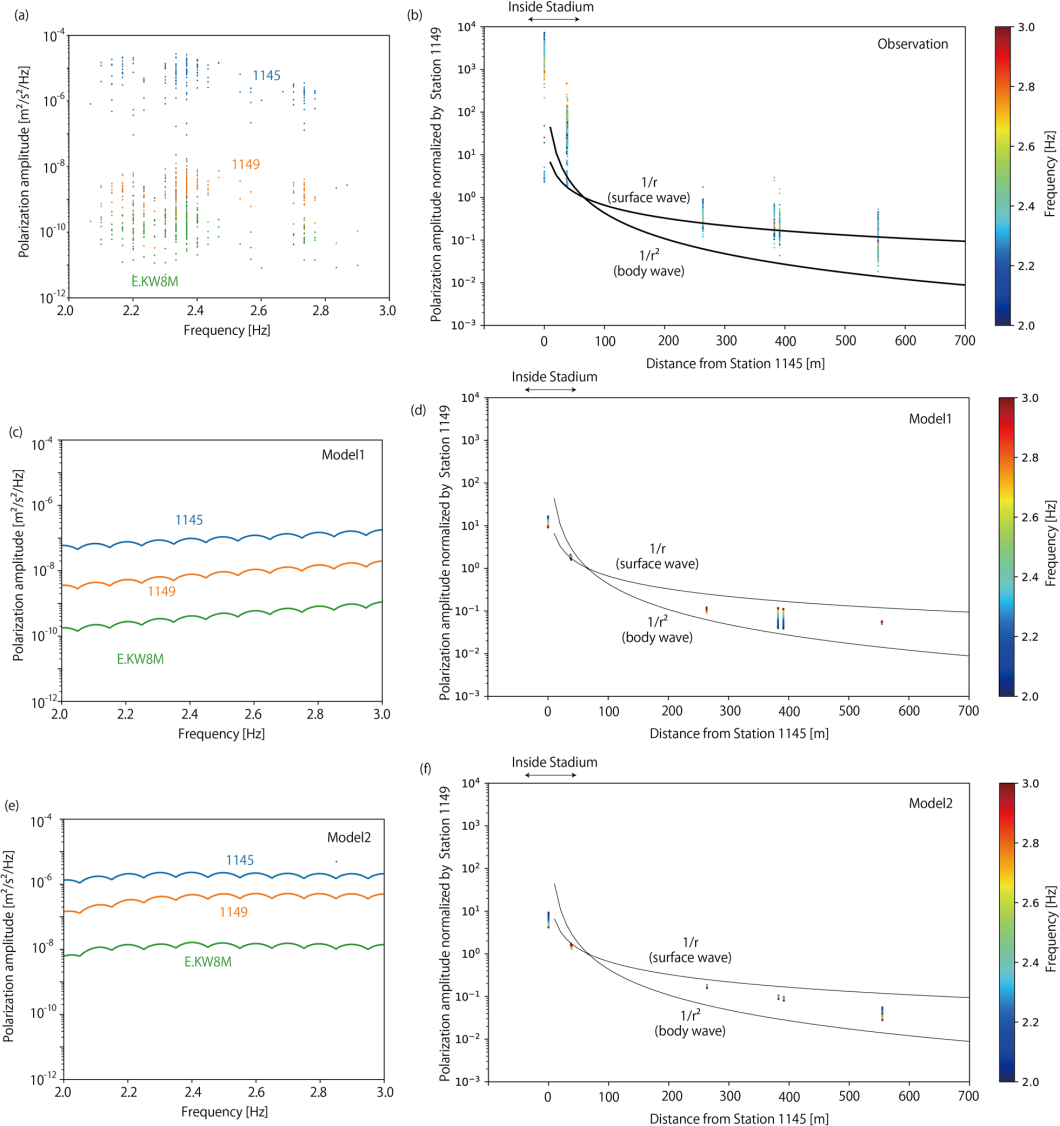
Scatter plots showing amplitude decay of the observed and synthetic seismic wave. **a** Polarization amplitudes from observation for three stations (Station 1145 in blue, 1149 in orange, and E.KW8M in green) are plotted against characteristic frequency. **b** Polarization amplitudes from observation for all stations are plotted against distance from Station 1145, which is almost the same location as the center of energetic supporters. Amplitudes are normalized by amplitude at Station 1149. Colors of dots represent characteristic frequencies of seismic waves. Black curves represent r^−1^ and r^−2^ decay, which corresponds to surface waves and body waves, respectively, for references. **c**, **d** Show synthetic results for Model 1 same as in **a**, **b**, respectively. **e**, **f** Show synthetic results for Model 2 same as **a**, **b**, respectively

**Fig. 10:**
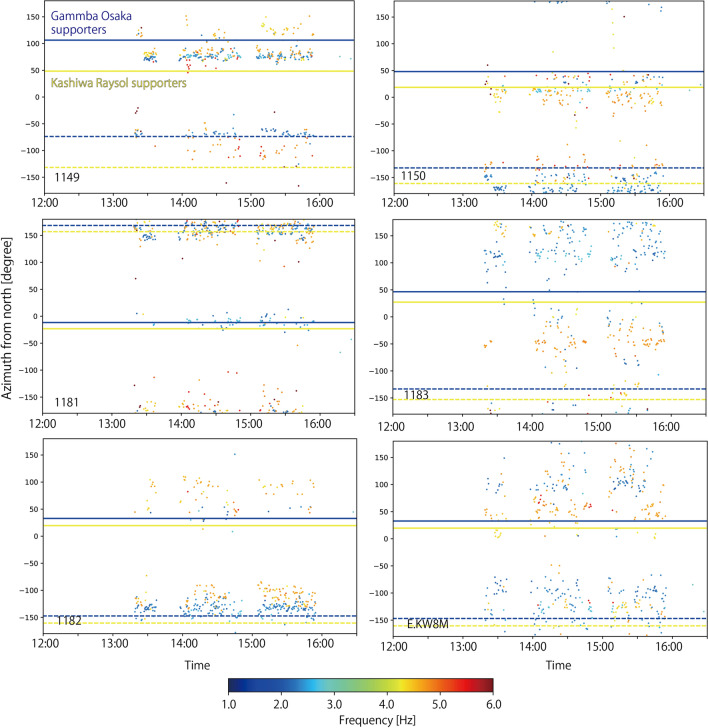
Time plots of polarization azimuths at stations located outside the stadium. Colors of dots represent characteristic frequencies. Solid yellow and blue horizontal lines are the azimuth toward supporters for Kashiwa Reysol and Gamba Osaka in the stadium, respectively. Dashed yellow and blue horizontal lines are the 180º opposite azimuths toward supporters for Kashiwa Reysol and Gamba Osaka in the stadium, respectively

Next, we investigated the amplitude decay and polarization azimuths of the seismic waves. Figure 9a, b shows the spatial decay of the polarization amplitude. We selected polarization amplitudes with the obtained characteristic frequencies within the same time window. Figure 9a shows the polarization amplitude observed at the three stations as a function of the characteristic frequency between 2 and 3 Hz, which corresponds to the lowest characteristic frequency band excited by jumping. The polarization amplitude was large (~ 10^–5^ m^2^/s^2^/Hz) at Station 1145, which is inside the stadium. However, it is much smaller (10^–9^ – 10^–8^ m^2^/s^2^/Hz) at Station 1149, which is located just outside the stadium. It further decayed to ~ 10^–10^ m^2^/s^2^/Hz at the MeSO-net station E.KW8M. We did not observe a clear frequency dependence in the polarization amplitude. Figure 9b shows the amplitude decay as a function of the distance *r* from Station 1145, which is almost the same location as the center of the energetic supporters. In the figure, amplitudes are normalized by those of the same time window at station 1149. Outside the stadium, the amplitude decays at *r*^−1^. We cannot observe clear trends in the differences in the amplitude decay for different characteristic frequencies. Figure 10 shows the calculated polarization azimuths. At some stations (1150, 1181), polarization azimuths are consistent with the azimuths where supporters of Kashiwa Reysol and Gamba Osaka are located. However, at other stations, the polarization azimuths differed from the azimuths of the supporters.

**Fig. 11:**
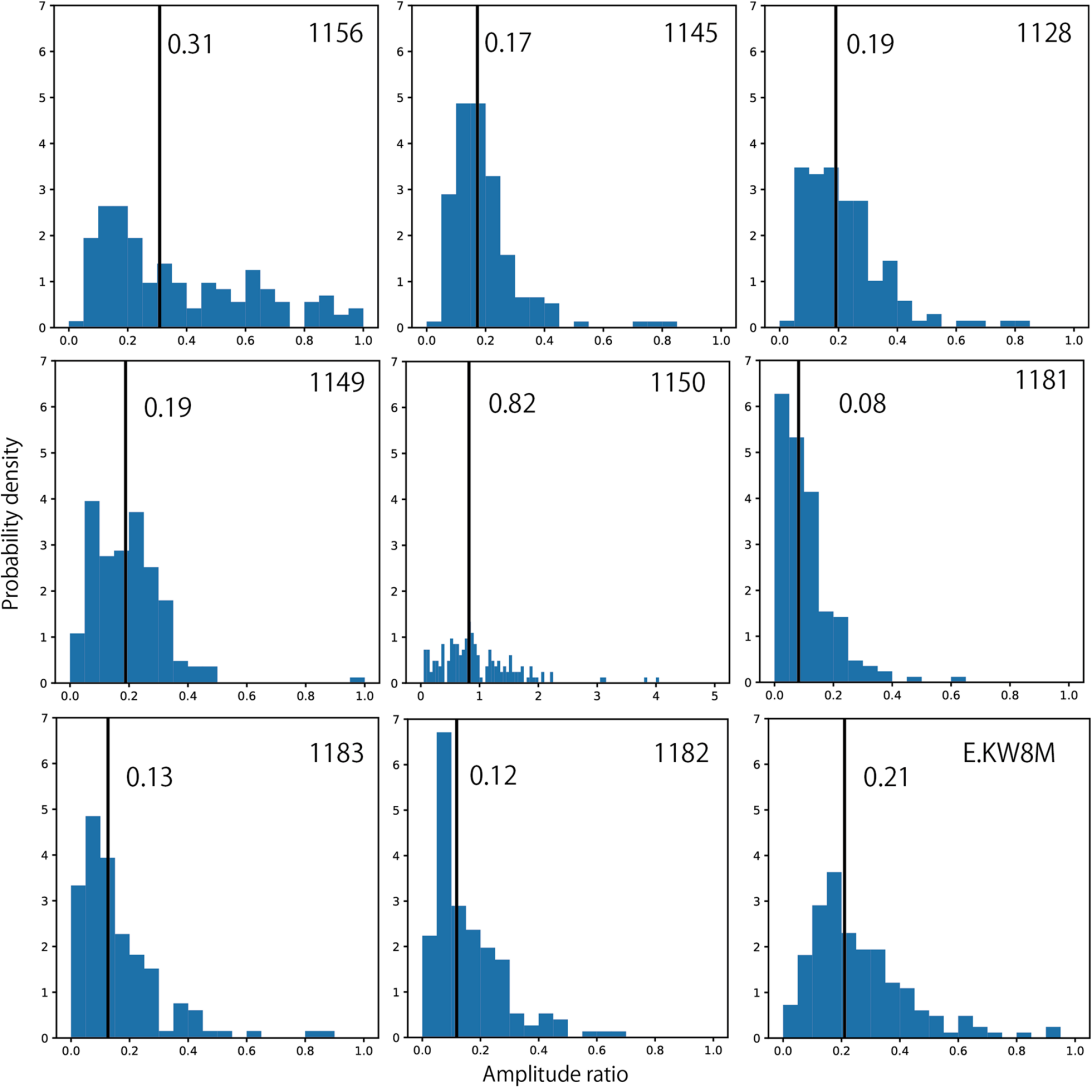
Histograms illustrating the amplitude ratio of seismic waves with two-times characteristic frequency of chants rhythms against those with the lowest characteristic frequency. Vertical black lines represent median values in distributions

Lastly, we checked the polarization amplitude ratio of seismic waves with twice the frequency of chant rhythms against those with the frequencies of chant rhythms. If we neglect the difference in seismic attenuation through propagation owing to different frequencies, the ratio corresponds to the ratio of $$\left( {{\raise0.7ex\hbox{${c_{2} }$} \!\mathord{\left/ {\vphantom {{c_{2} } {c_{1} }}}\right.\kern-\nulldelimiterspace} \!\lower0.7ex\hbox{${c_{1} }$}}} \right)^{2}$$ in the seismic source model (Eq. 1). When many people jump together, dynamic loading factors decrease with the number of people due to their incoherent actions (Ellis and Ji 2004). Although such data can be obtained through experiments with a relatively small number of people (less than 100), experiments with many people are difficult. Our observations can be regarded as experiments involving thousands of people. Figure 11 shows histograms of the polarization amplitude ratios. Median values are about 0.1 – 0.3 for all stations except for Station 1150. Ellis and Ji (2004) obtained regression lines for the dynamic loading factor as a function of the number of jumping people as follows:

2 $${c}_{1}=1.61 \times {{N}_{p}}^{-0.082},$$

3 $${c}_{2}=0.94 \times {{N}_{p}}^{-0.24},$$

where *N*_*p*_ is the number of people. Using Eqs. (2) and (3), we obtained an amplitude ratio $$\left( {{\raise0.7ex\hbox{${c_{2} }$} \!\mathord{\left/ {\vphantom {{c_{2} } {c_{1} }}}\right.\kern-\nulldelimiterspace} \!\lower0.7ex\hbox{${c_{1} }$}}} \right)^{2}$$ of 0.04, assuming *N*_*p*_ of 1000. The observed amplitude ratios were one order magnitude larger than this value. This may reflect a more efficient synchronization of the actions of the people in the stadium than in the experiments. The observed amplitude ratio at Station 1150 was even larger than that at other stations, whose reason is not clear.

## Synthetic wave calculation

**Fig. 12:**
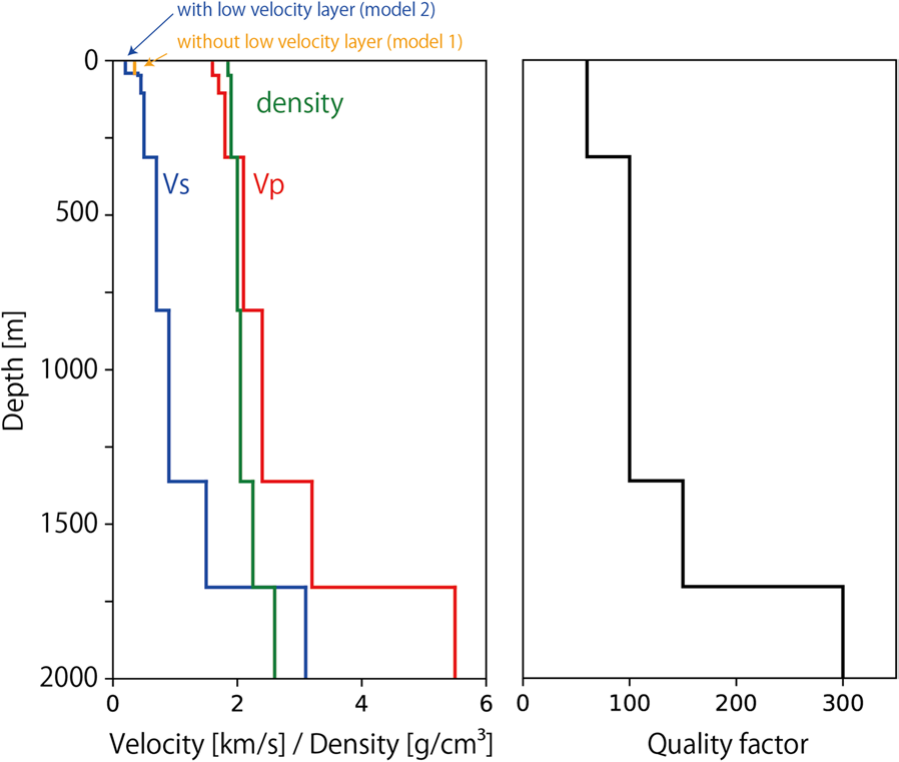
The structure of the physical properties based on JSHIS, assumed for synthetic wave calculations. Model 1 and Model 2 are the same except for S-wave velocity at a top layer

In this section, we calculate the synthetic waveforms generated by J. LEAGUE supporters to compare them with the observations. We assumed a semi-infinite medium with a one-dimensional velocity structure and referred to J-SHIS for shallow subsurface structures in this region (Fujiwara et al. 2009, 2012; National Research Institute for Earth Science and Disaster Resilience 2019). Figure 12 shows the assumed structures of the physical properties. We prepared two models based on the J-SHIS database for this region. Model 1 follows the deep subsurface structure of J-SHIS. Model 2 is based on the combined shallow and deep layer model. Model 2 followed Model 1 with an additional low-velocity (200 m/s) layer at the top, which was based on the average S-wave velocity for the top 30 m depth in this region estimated by J-SHIS (Wakamatsu and Matsuoka 2013). The seismic sources of supporters were assumed to be downward point sources located at the center of the stand for Kashiwa Reysol supporters (Fig. 1). Synthetic velocity waveforms generated by impulsive downward vertical single force on the ground surface were calculated using “computer programs in seismology” (Herrmann 2013).

Figure 8b and 8c shows the calculated synthetic waveforms for pulsive single force of the two models. In Model 1, the wave packets travel at approximately 300 – 400 m/s. Dispersion of the waveforms are weak, and all phases are distributed between lines of 300 m/s and 400 m/s. In Model 2, the traveling wave packets show significant dispersion. The largest amplitude travels at approximately 200 m/s. though the onset of waveforms travels at approximately 300 m/s. Later phases slower than 200 m/s are also evident. Comparing these synthetics with observations, dispersive features of the observed waveforms are similar to synthetics of Model 2. The travel time of the largest amplitude in Model 2 is also similar to observations at stations 1150, 1182, and E.KW8M, though faster onsets at stations 1181 and 1182 are not explained. More detailed modeling of the subsurface structure will be necessary to fully explain the observation.

Next, we calculated the synthetic waveforms generated by jumping of many people. We assume that 1000 people each with 65 kg weight synchronizing jump together. Periods of jumping are tested between 2.0 Hz and 3.0 Hz with increments of 0.01 Hz. The source-time function is a sinusoidal curve with an assumed period. The source-time functions were convolved to calculated synthetic waveforms. Polarization analysis was applied to the calculated sinusoidal waveforms. Figure 9c–f shows the amplitude decay of the calculated waveforms. In Model 1, the polarization amplitudes at Station 1149 and E.KW8M were comparable to those of the observations (Fig. 9a). However, the polarization amplitude at Station 1145 was much smaller than that observed. Although we assumed a point source, people were distributed throughout the stand. As Station 1145 was inside the stadium, a much larger amplitude should be observed because of the finiteness of the seismic source. We also observed a clear frequency dependence of the polarization amplitudes, which was not observed in the observations. The polarization amplitude decays with the distance between r^−1^ and r^−2^ (Fig. 9d), which is similar to those of the observations (Fig. 9b). In model 2, we did not observe a frequency dependence in polarization amplitudes, which is consistent with those of the observations (Fig. 9e). The polarization amplitudes at Station 1149 and E.KW8M are larger than the observations. Although we assume perfect synchronization of jumping among 1000 people, the real situation is different, which will reduce the effective size of seismic sources. The polarization amplitude decays with the distance between r^−1^ and r^−2^ (Fig. 9f), which is similar to those of the observations (Fig. 9b).

The relation between amplitudes of seismic waves and the number of jumping people is another good example of monitoring collective human activities through seismic waves. In this calculation, we assumed perfect synchronization of supporters’ jump. However, there should be differences in jumping periods and phases among supporters. How it statistically distributes will affect excitation efficiency of seismic waves. Information on traveling paths of seismic waves dependent on three-dimensional subsurface heterogeneity and complex topography in this region is also important because they control attenuation of seismic waves. With such further studies, it will become possible to estimate the number of jumping supporters based on seismic waves.

As we assume a one-dimensional velocity structure of the semi-infinite medium, the polarization direction of the synthetic waves is directed toward the seismic sources. This could not explain the observed complex polarity azimuths (Fig. 10). According to Geospatial Information Authority of Japan, there are about 10 m elevation differences between the stadium and southern residential area. Such complex topography in this region could bend the traveling paths of seismic waves dependently on frequencies. To fully explain our observations, we should consider such topography and small-scale structural heterogeneities in this region.

## Conclusions

This study conducted temporary seismic observations targeting J. LEAGUE supporters who act as unique seismic sources in urban environments. The observed seismic signals have characteristic frequencies that exactly match the rhythms of the chants that the supporters are singing. This confirms that the collective action of jumping supporters during the game generates seismic waves. Characteristic frequencies in seismic records are integer multiples of jumping rhythms. This agrees with the loading model of jumping people suggested in an earlier study. Observing J. LEAGUE supporters is useful for validating such models in which thousands of people are acting together. It is also a good example that we can monitor collective human activities (i.e., distinguishing which chant supporters are singing or estimating the number of jumping supporters) based on seismic signals. As various human activities induce seismic signals, further studies will be necessary to fully understand relations between seismic signals and what we want to monitor through seismic signals, though our results suggest that such monitoring in urban environments is possible. Seismic data would have fewer potential privacy concerns than other types of data on human activities. In addition, seismic data are expected to include integral information on various human activities, including economic activity, mobility, and various collective actions of people. Therefore, seismic data would be a useful tool to monitor human activities.

The observed travel time and amplitude decay are consistent with the synthetic waveforms calculated based on the subsurface structure of the J-SHIS. Comparing the two models with and without a low-velocity layer at the ground surface, the observed dispersive features of traveling waveforms are similar to the synthetic calculations for the model with a low-velocity layer. Travel times of the observed waveforms are also partly consistent with the model with a low-velocity layer. This suggests that the subsurface structure constructed using J-SHIS is good in this region. However, a part of the polarization direction cannot be reproduced by a one-dimensional structure. The local topography and detailed structural heterogeneity can be important for explaining the polarization azimuths. Observations of unique seismic sources in urban environments, such as earth-shaking J. LEAGUE supports, also have the potential to improve local subsurface structural models. As such human-induced seismic signals are energetic and predictable, seismic observations for structural investigations can be conducted more efficiently using them than using earthquake signals or ambient noises. In addition, such human-induced seismic signals could be everywhere in the urban environment. Therefore, we suggest that human-induced seismic signals would be useful as an artificial seismic source in many cities for structural investigations.

## Data Availability

Data used in this study are available from the corresponding author upon reasonable request.

## Abbreviations

J-SHIS: Japan Seismic Hazard Information Station
MeSO-net: Metropolitan Seismic Observation Network
